# Salivary and serum haptoglobin, adenosine deaminase, and immunoglobulin G in growing pigs

**DOI:** 10.1186/s40813-024-00368-8

**Published:** 2024-05-21

**Authors:** Virpi Piirainen, Ana M. Gutiérrez, Mari Heinonen, Emilia König, Anna Valros, Sami Junnikkala

**Affiliations:** 1 Faculty of Veterinary Medicine, Department of Production Animal Medicine, University of Helsinki, Leissantie 41, Saarentaus, FI-04920 Finland; 2 Department of Animal Medicine and Surgery, BioVetMed Research group, University of Murcia, Campus de Espinardo, Murcia, 30100 Spain; 3 Research Centre for Animal Welfare, Faculty of Veterinary Medicine, University of Helsinki, Koetilantie 7, Helsinki, FI-00790 Finland; 4 Faculty of Veterinary Medicine, Department of Veterinary Biosciences, University of Helsinki, Agnes Sjöbergin katu 2, Helsinki, FI-00790 Finland

**Keywords:** Biomarker, Correlation, Field study, Gender, Longitudinal, Porcine, Pig farm, Saliva, Serum

## Abstract

**Background:**

Identification of animals in need of medical treatment is important in porcine health management, where analytical samples applicable at farm level could be utilized. Several biomarkers are measurable in saliva, which is less stressful to collect than blood. Saliva sampling is easy to learn and repeatable, making it suitable for monitoring purposes. Previous research suggests that porcine health biomarkers are dependent on production stage and gender, and that combining biomarkers improves diagnostic sensitivity. However, proper monitoring of biomarkers during the complete production cycle has not been studied. We aimed to describe the dynamics of salivary and serum haptoglobin (Hp), adenosine deaminase (ADA), and immunoglobulin G (IgG) in four production stages (suckling, early growing, late growing, finishing), on commercial Finnish pig farms using a total of 117 piglets. The relationship between gender and biomarker dynamics was investigated, as well as the relationships between these biomarkers in saliva and serum.

**Results:**

The highest salivary concentrations of Hp, ADA and IgG were measured in suckling piglets. The differences between production stages were generally larger in saliva than for the corresponding serum biomarkers. All correlation coefficients between salivary biomarkers were positive in each production stage and the strength of the correlation varied from 0.245 to 0.762. No similar trend was observed regarding correlation coefficients either between serum biomarkers or between salivary and serum biomarkers. Gender was associated with some biomarker concentrations.

**Conclusions:**

The biomarker dynamics supported previous findings that collection of analytical samples should be conducted in age-matched populations. Positive and even strong relationships between salivary biomarkers indicate the potential to use especially saliva for health monitoring. Our results also suggest the importance of considering gender effects when assessing some salivary or serum biomarkers.

**Supplementary Information:**

The online version contains supplementary material available at 10.1186/s40813-024-00368-8.

## Background

Monitoring of health, disease, and related production parameters, such as pig growth, is a prerequisite for successful health management in pig farming. It is necessary to develop methods for more accurate evaluation of the health status of pigs, and clinical analytics in body fluids could be utilized as part of this. Saliva sampling offers a minimally invasive and less stressful alternative to blood sampling [1], and several biomarkers can be measured in porcine saliva [2, 3]. Additionally, saliva collection is quick, suitable for repeatable measurements and applicable at both the group [4] and individual level [5]. Saliva collection is easy to learn [1], which is why it can be implemented by farm employees, also making it cost-effective.

Acute phase proteins (APPs) have been utilized in veterinary medicine for decades and recognized as non-specific biomarkers of illness [6]. Infection, inflammation, or trauma can trigger a pro-inflammatory response, which mainly stimulates APP production in the liver, from which APPs enter the blood circulation [6]. Pig studies under field conditions have demonstrated that in case of natural infection, the acute phase response is stronger in animals having clinical signs of disease or infected with multiple pathogens than sub-clinically infected animals, or those with infections caused by a single pathogen [6]. However, being very sensitive, APPs respond to an inflammatory stimulus before clinical signs appear, which supports the use of APPs as a marker of subclinical conditions [7]. Therefore, a combination of APPs [3, 8] or the combination of APPs with other health biomarkers [3, 9] has been suggested to improve the health evaluation of pigs.

Adenosine deaminase (ADA) is an immunomodulatory enzyme that is distributed in most cells and tissues [10], and it has a proposed role in the function of cell-mediated adaptive immunity in humans [11, 12]. Moreover, the serum immunoglobulin concentration can be used to assess the activation of adaptive humoral immunity [13], with immunoglobulin G (IgG) being a biomarker of systemic immune activation [14]. In particular, the IgG concentration is predominantly elevated in the case of an infectious disease [7]. The measurement of total IgG may have clinical value, since IgG seems more sensitive than other immunoglobulin-isotypes to differentiate between healthy and diseased pigs [15]. When measured from the newborn piglets’ serum, the IgG level also reflects the amount of maternal IgG transferred to piglets via colostrum [13, 14]. Immunoglobulin G in human saliva is primarily derived from serum (mainly via gingival crevices) [16]. By analyzing serum IgG of growing pigs, we wanted to evaluate the passive immunity of suckling piglets, evaluate the humoral immune status of pigs during later production stages and to compare IgG concentrations between saliva and serum.

Some authors have reported that the diagnostic sensitivity of saliva is better than that of serum for pig health evaluation [3, 9]. Furthermore, previous research has shown that the dynamics of some porcine health biomarkers is dependent on age [for serum Hp, see 17; for salivary Hp, see 18, 19; for serum IgG see 20 and on gender see 16–18].

In the present study, we aimed to describe the dynamics of salivary and serum Hp, ADA, and IgG of growing pigs in four production stages (suckling, early growing, late growing and finishing), on commercial Finnish pig farms. We also investigated the association between gender and biomarker dynamics and the correlations of abovementioned biomarkers in saliva and serum at different ages during growing.

## Results

Altogether 35, 22, 26, and 34 pigs were included in the farm pairs 1–4, respectively. Birth weight of the study pigs was on average 1.4 kg (SD 0.3 kg), being 6.8 kg (SD 1.4 kg), and 22.2 kg (SD 6.2 kg) at early and late growing stages. The average finishing pig weight, measured at the end of finishing stage, was 110.1 kg (SD 19.7 kg). No statistical differences were found in the weight between female and male pigs at any of the production stages.

### Dynamics of biomarkers in saliva and serum in growing pigs

Concentrations of salivary and serum biomarkers (based on raw data) are summarized in Tables 1 and 2, and the outcomes from the final models are reported in detail in Supplementary Tables 1a–f (Additional File 1).

Suckling piglets had the highest measured salivary concentration of all biomarkers. Salivary Hp concentration decreased until the end of finishing stage (Table 1), and according to a repeated-measures linear mixed model (LMM), differed between production stages (F_3,133_ = 250, *p* < 0.001, pairwise comparisons, *p* < 0.001 between all stages except for between early growing and late growing stages (*p* = 0.11). Salivary ADA concentration also differed between production stages (F_3,123_ = 51, *p* < 0.001). Salivary ADA concentrations in suckling piglets were higher than in all other production stages (pairwise comparisons, *p* < 0.001); at early growing stage differed from those at suckling and at late growing stages (*p* < 0.001 for both) and tended to differ from those of finishing stage (*p* = 0.07). Salivary ADA concentrations at late growing and finishing stages did not differ from each other (*p* > 0.1). Salivary IgG concentrations showed similar dynamics as salivary ADA (Table 1), and production stage was a significant predictor of the salivary IgG concentration (F _3,105_ = 79, *p* < 0.001). All production stages differed regarding their salivary IgG concentration (pairwise comparisons, *p* < 0.001–0.02).

**Table 1:** Dynamics of three salivary biomarkers of pigs in four production stages

	Suckling ^2)^ Age 4 (1–5) days	Early growing Age 24 (21–33) days	Late growing Age 66 (61–80) days	Finishing Age 165 (132–168) days
Hp (µg/mL), median IQ	8.24 4.74	1.97 1.78	1.07 2.26	0.36 0.40
n	56	78	68	78
ADA (U/L)^1)^, median IQ	894.58 400.96	606.61 405.29	371.30 301.30	552.61 265.31
n	75	85	73	77
IgG (µg/mL), median IQ	504.71 612.55	37.76 83.61	13.45 27.44	13.83 11.99
n	38	83	72	78

Hp = haptoglobin, ADA = adenosine deaminase, ^(1)^ in 1:16 dilution, IgG = immunoglobulin G. Age is presented as median (minimum-maximum) days. ^(2)^ Male piglets were intact at the time of sampling. Descriptive statistics are presented as raw values. IQ = interquartile range. n = number of samples (differences in numbers are due to differences in the available analysis results for separate biomarkers in each stage of production)

The concentration of serum Hp was highest at late growing phase (Table 2) and the production stage predicted the concentration (F_3,183_ = 7.2, *p* < 0.001). Pairwise comparisons showed that only production stages early and late growing (*p* = 0.01) and production stages late growing and finishing differed (*p* < 0.001) from each other. Both serum ADA and serum IgG were associated with production stage (F_3,214_ = 9.5 and F_2, 195_ = 246, respectively; *p* < 0.001 for both). The highest serum ADA concentration was measured at early growing stage (Table 2), being significantly higher compared to all other production stages (pairwise comparisons, *p* < 0.001). The estimated serum IgG concentration in suckling piglets was higher than in any other production stage (Table 2). The actual serum IgG concentration was determined from the beginning of growing stage onwards, from which stage the concentration increased towards the finishing stage (pairwise comparisons, *p* < 0.001 between all).

**Table 2:** Dynamics of three serum biomarkers of pigs in four production stages

	Suckling ^1)^ Age 4 (1–5) days	Early growing Age 24 (21–33) days	Late growing Age 66 (61–80) days	Finishing Age 165 (132–168) days
Hp (mg/mL), median IQ	0.36 0.92	0.16 0.75	0.83 1.21	0.37 0.56
n	93	117	114	113
ADA (U/L), median IQ	506.62 168.65	589.94 196.65	489.95 191.31	456.62 156.65
n	95	117	115	113
IgG (mg/mL), median IQ	44.74^2)^ 13.09	5.08 2.60	5.95 2.86	10.03 3.28
n	103	115	115	114

Hp = haptoglobin, ADA = adenosine deaminase, IgG = immunoglobulin G. Age is presented as median (minimum-maximum) days. ^(1)^ Male piglets were intact at the time of sampling, ^(2)^ Estimated concentration in suckling piglets, which corresponds to a serum immunoglobulin ratio of 0.13 (0.04). Descriptive statistics are presented as raw values. IQ = interquartile range. n = number of samples (differences in numbers are due to differences in the available analysis results of for separate biomarkers in each stage of production)

The median concentrations of all biomarkers in both sample types are presented separately for males and females in supplementary material (Table 2a for saliva and 2b for serum, Additional File 1). According to the LMM with repeated measures, only the salivary ADA concentration was dependent on gender (F_1,254_ = 5.6, *p* = 0.018), females having lower concentration than males. Gender tended to influence serum IgG (F_1,29_ = 3.6, *p* = 0.06), but interacted significantly with age (F_2,195_ = 5.0, *p* = 0.008). No similar results were found regarding other salivary biomarkers, or any of the serum biomarkers (repeated-measures LMM, *p* > 0.05). Pig weight was a significant predictor in the repeated-measures LMM for only salivary ADA (F_1,86_ = 5.0, *p* = 0.02) and salivary IgG (F_1,80_ = 8.6, *p* = 0.004). For both salivary ADA (Coefficient = 2.3, 95% CI 0.26–4.37) and salivary IgG (Back-transformed Coefficient = 1.01, 95% CI 1.00-1.02), the connection was positive.

### Correlations between the studied biomarkers in four production stages

Overall, all associations between salivary biomarkers were positive in each production stage, according to the coefficient of correlation. The strength of the correlations varied from very weak to strong, and significant correlations were found in all production stages except suckling. All correlation coefficients between serum biomarkers, as well as between salivary and serum biomarkers, were very weak or weak. Moreover, both positive and negative correlation coefficients were observed. Correlation coefficients are detailed for each production stage in Tables 3, 4, 5, 6.

**Table 3:** Spearman correlations between three salivary and serum biomarkers in suckling piglets (median age 4, 1–5 days)

	Hp, Saliva	ADA, saliva	IgG, saliva	Hp, serum	ADA, serum	Immunocrit
Hp, saliva		ρ 0.08 P 0.583 n 56	ρ 0.151 P 0.373 n 37	ρ -0.113 P 0.450 n 47	ρ 0.114 P 0.432 n 50	ρ 0.179 P 0.214 n 50
ADA, saliva			ρ 0.113 P 0.500 n 38	ρ 0.028 P 0.854 n 47	ρ -0.218 P 0.088 n 62	ρ 0.009 P 0.942 n 67
IgG, saliva				ρ 0.314 P 0.144 n 23	ρ -0.279 P 0.104 n 35	ρ 0.037 P 0.835 n 35
Hp, serum					ρ -0.132 P 0.212 n 91	**ρ -0.301** **P 0.003** **n 93**
ADA, serum						**ρ 0.333** *P* < 0.001 **n 95**

Salivary haptoglobin (Hp) and immunoglobulin G (IgG) measured as µg/mL, serum Hp and IgG as mg/mL, adenosine deaminase (ADA) in both body fluids as U/L. Immunocrit = serum immunoglobulin ratio. ρ = Spearman correlation coefficient, P = p-value, n = number of comparisons (differences in numbers are due to differences in the available paired analysis results for separate biomarkers). Significant relationships at the significance level *p* < 0.05 are bolded

**Table 4:** Spearman correlations between three salivary and serum biomarkers at early growing stage (median age 24, 21–33 days)

	Hp, saliva	ADA, saliva	IgG, saliva	Hp, serum	ADA, serum	IgG, serum
Hp, saliva		**ρ 0.284** **P 0.014** **n 75**	**ρ 0.391** *P* < 0.001 **n 76**	**ρ 0.359** **P 0.001** **n 78**	ρ -0.042 P 0.713 n 78	ρ -0.205 P 0.074 n 77
ADA, saliva			**ρ 0.315** **P 0.004** **n 82**	ρ 0.021 P 0.852 n 85	ρ -0.077 P 0.486 n 85	ρ -0.046 P 0.677 n 83
IgG, saliva				ρ -0.198 P 0.073 n 83	ρ 0.138 P 0.212 n 83	**ρ 0.245** **P 0.027** **n 81**
Hp, serum					ρ -0.117 P 0.210 n 117	ρ -0.008 P 0.931 n 115
ADA, serum						ρ -0.115 P 0.222 n 115

Salivary haptoglobin (Hp) and immunoglobulin G (IgG) measured as µg/mL, serum Hp and IgG as mg/mL, adenosine deaminase (ADA) in both body fluids as U/L. ρ = Spearman correlation coefficient, P = p-value, n = number of comparisons (differences in numbers are due to differences in the available paired analysis results for separate biomarkers). Significant relationships at the significance level *p* < 0.05 are bolded

**Table 5:** Spearman correlations between three salivary and serum biomarkers of pigs at late growing stage (median 66, 61–80 days)

	Hp, saliva	ADA, saliva	IgG, saliva	Hp, serum	ADA, serum	IgG, serum
Hp, saliva		**ρ 0.522** *P* < 0.001 **n 68**	**ρ 0.762** *P* < 0.001 **n 72**	ρ 0.237 P 0.056 n 66	ρ 0.039 P 0.757 n 67	ρ 0.117 P 0.347 n 67
ADA, saliva			**ρ 0.627** *P* < 0.001 **n 72**	ρ -0.101 P 0.403 n 71	ρ -0.218 P 0.066 n 72	ρ 0.191 P 0.108 n 72
IgG, saliva				ρ -0.132 P 0.276 n 77	ρ -0.096 P 0.426 n 71	**ρ 0.252** **P 0.034** **n 71**
Hp, serum					ρ 0.153 P 0.105 n 114	ρ 0.095 P 0.312 n 114
ADA, serum						**ρ -0.275** **P 0.003** **n 115**

Salivary haptoglobin (Hp) and immunoglobulin G (IgG) measured as µg/mL, serum Hp and IgG as mg/mL, adenosine deaminase (ADA) in both body fluids as U/L. ρ = Spearman correlation coefficient, P = p-value, n = number of comparisons (differences in numbers are due to differences in the available paired analysis results for separate biomarkers). Significant relationships at the significance level *p* < 0.05 are bolded

**Table 6:** Spearman correlations between three salivary and serum biomarkers at finishing stage (165, 132–168 days)

	Hp, saliva	ADA, Saliva	IgG, saliva	Hp, serum	ADA, serum	IgG, serum
Hp, saliva		ρ 0.150 P 0.194 n 77	**ρ 0.614** *P* < 0.001 **n 78**	**ρ 0.340** **P 0.003** **n 76**	ρ 0.213 P 0.064 n 76	**ρ -0.252** **P 0.027** **n 77**
ADA, saliva			**ρ 0.316** **P 0.005** **n 77**	ρ -0.122 P 0.294 n 76	ρ -0.022 P 0.853 n 76	**ρ 0.258** **P 0.025** **n 76**
IgG, saliva				ρ -0.020 P 0.865 n 76	ρ 0.178 P 0.124 n 76	ρ 0.133 P 0.249 n 77
Hp, serum					ρ 0.086 P 0.365 n 113	**ρ -0.188** **P 0.046** **n 113**
ADA, serum						ρ 0.142 P 0.135 n 113

Salivary haptoglobin (Hp) and immunoglobulin G (IgG) measured as µg/mL, serum Hp and IgG as mg/mL, adenosine deaminase (ADA) in both body fluids as U/L. ρ = Spearman correlation coefficient, P = p-value, n = number of comparisons (differences in numbers are due to differences in the available paired analysis results for separate biomarkers). Significant relationships at the significance level *p* < 0.05 are bolded

## Discussion

In the present study, we selected Hp, ADA and IgG for analytes that could indicate activation of innate immunity (Hp), adaptive cell-mediated immunity (ADA) and adaptive humoral immunity (IgG) in growing pigs. Only limited studies compare the concentrations of these biomarkers between saliva and serum [3, 7], and none in which they all (Hp, ADA, IgG) are analyzed together. Significant differences in salivary biomarker concentrations were found between production stages. The highest salivary concentrations of Hp, ADA and IgG were measured in suckling piglets. Haptoglobin has previously been determined in sow colostrum [17], which in this case could be considered a possible factor behind the high Hp concentration measured in the saliva of suckling piglets. On the other hand, salivary Hp could be produced locally by salivary glands [18]. We found approximately 1000-fold lower concentrations of Hp in saliva than in serum, what is in line with other studies [3, 15, 19]. High concentrations of salivary ADA in suckling piglets may be linked to immune development in early life, which is supported by human studies: ADA was reported to influence lymphocyte development [10] and it probably regulates T-cell responses towards the anti-inflammatory Th2 direction during the first months of life in humans [12]. High ADA concentration in suckling pig saliva raised the question if that was of maternal origin. However, when we analyzed some sow colostrum samples to answer this question, they did not contain detectable amounts of ADA (data not shown). Porcine salivary IgG has only rarely been measured [for example, see 15], and no longitudinal data are available, thus preventing a comparison with previous pig studies.

From suckling towards the finishing stage, a decreasing trend was observed for salivary Hp, and until the late growing stage for ADA and IgG. Previous pig studies conducted under commercial conditions have reported similar decreasing trends regarding salivary Hp between 4 and 10 weeks of age [20], between 11 and 17 weeks of age [20], and recently for salivary Hp and ADA from birth until the finishing stage among clinically healthy pigs [21]. The authors suggested that the growth of the pigs and changes in their feeding could influence the concentrations of biomarkers [21].

Overall, smaller differences were found in serum concentrations compared to saliva for all three biomarkers. Similar results to ours, of no marked differences over time with respect to serum Hp have also been presented previously, when an acute phase response to different viruses was followed [20]. Piñeiro et al. [22], however, reported contradictory results under commercial conditions, as they found significant changes in serum Hp concentrations in healthy pigs from the age of four to twenty weeks. We measured the highest serum ADA concentration at early growing, and thereafter it decreased continuously towards the finishing stage. Proper interpretation of the serum ADA concentration is difficult, because it has not been measured longitudinally in other pig studies. For serum IgG, the dynamics were opposite to those for serum ADA. Serum IgG in suckling piglets is of maternal origin [13] and piglets therefore have high serum IgG concentrations during the first days of life [23]. In suckling pigs, we determined the serum immunoglobulin ratio, which has been shown to be a valid method for evaluating the passive transfer of maternal immunoglobulins from sows to piglets [24]. The degradation of maternal IgG molecules in piglets starts rapidly, resulting in a drop in the serum IgG concentration, and the lowest concentration can be detected at around four weeks of age [23]. The dynamics of serum IgG in the present study are consistent with what has been reported to be physiological for pigs [23, 25]. Human studies have shown that saliva IgG is primarily derived from serum mainly via gingival crevices [16]. We found high levels of IgG also in saliva of suckling pigs, demonstrating that saliva IgG is mainly derived from blood and is of maternal origin.

We observed that gender had an influence on the concentrations of some biomarkers. Previous studies [21, 26, 27] have demonstrated that gender has an impact on porcine salivary Hp and ADA dynamics. Sánchez et al. [26] reported a decreasing concentration of salivary Hp and ADA in intact male pigs from post-weaning to the finishing stage, while the opposite trend was observed for female pigs. Gutiérrez et al. [27] reported significantly higher salivary Hp and ADA concentrations in females compared to males in the finishing stage. Regarding IgG in saliva, the dynamics across genders resembled those observed regarding Hp and ADA, except for the suckling stage, when females had a two-fold higher median salivary IgG concentration compared to males (See Supplementary Table 2a, Additional File 1). Overall, studies reporting gender differences are difficult to compare, as the age and reproductive status of the studied individuals have varied. Sexual hormonal interference has been hypothesized [27], but there are clearly still other factors, such as management conditions or breed [22] that could influence in the dynamics of biomarkers through the production phases.

We found that all relationships between salivary biomarkers were positive and compared to serum, the strength of correlation was higher overall. A consistent finding was that the correlation between salivary Hp and IgG was moderate or strong through three production stages, excluding suckling piglets. Moreover, salivary ADA correlated with salivary Hp at early and late growing stages, and in the latter stage also correlated with salivary IgG. Previous studies have reported a positive correlation between salivary Hp and ADA in finishing pigs [3, 28]. The positive correlations observed could be explained by the connection of the three biomarkers to the immune system. However, the lack of strong correlations in the present study could be due to the different immune functions of the biomarkers: ADA [11, 12] and IgG [13] are related to the adaptive immune system, while Hp is linked to the innate immune response [6].

Our results regarding the associations between serum biomarkers, or between saliva and serum biomarkers, were more ambiguous. This is in concordance with the study of Sánchez et al. [3] on finishing pigs. A significant negative correlation between serum Hp and the serum immunoglobulin ratio could not be related to passive immune acquisition, as a high serum immunoglobulin ratio in suckling piglets refers to successful colostrum intake [24] and serum Hp in suckling piglets is rapidly increased during the first 12 h with colostrum intake [17]. On the contrary, we found a significant positive relationship between serum ADA and the serum IgG ratio. One plausible explanation is that these parameters reflect a common function of adaptive cell-mediated [11, 12] and humoral [13] immunity.

The study population in the present study originated from four different farms, and the influence of diverging housing and management practices as well as of existing subclinical diseases could not therefore be ruled out. For example, farm-dependent differences in salivary [20] as well as serum [20, 22] Hp have been reported. Our aim, however, was to assess the dynamics and relationships of porcine biomarkers under field conditions that represents the real-life situation. Sanchez et al. [26] have reported an impact of pig breed on salivary biomarkers, and a genetic influence on the results cannot therefore be ruled out, as our study population consisted of genetically different pigs. Piglets with the lowest birth weight were excluded from the study because of their poor prediction of survival [29]. This decision was based on our aim to follow up the study pigs until slaughter. Under the prevailing conditions on the farms, we were not always able to collect saliva before blood sampling. However, we did not determine stress biomarkers per se, wherein which case the sampling order should have been considered more critically.

## Conclusions

Evident differences between production stages support previous findings that the collection of analytical samples should be conducted in age-matched populations. The role of high salivary ADA and Hp in the developing immune system would be interesting to explore further. Positive and even strong relationships between salivary biomarkers indicate the potential to use especially saliva as a diagnostic specimen, although our results further suggest that it is important to consider gender effects when assessing biomarkers as a tool for monitoring pig health.

## Methods

### Characteristics of the farms, animals, and management

Four commercial piglet-producing farms and their respective four finishing farms from western and south-western Finland were recruited for the study and formed four farm pairs (F1–F4). After birth, the pigs were grown on the same piglet-producing farms until they were moved to their respective finishing farms at an average age of ten weeks and target weight of 30 kg. The size of the farms (average number of sows and finishing pigs, respectively) was 940 and 2100 for F1, 450 and 680 for F2, 450 and 280 for F3, and 1100 and 2800 for F4.

The sow genetics were Topics Norsvin on F1 and F4, DanBred on F3 and crossbred DanBred x Landrace on F2. Pregnant sows were moved to farrowing rooms three (F2) or five (F1, F3, F4) days prior to the expected farrowing day. Sows were housed in farrowing crates in pens (4.6–4.8 m^2^) with partly slatted floors and no bedding. All sows were fed with a standard liquid meal. Male piglets were castrated before seven days of age and their castration pain was alleviated with a non-steroidal anti-inflammatory drug administered intramuscularly. All piglets received an intramuscular iron injection within the first week after birth and, except for F2, all piglets received one oral dose of anticoccidial medication. The teeth of the piglets were not clipped/ground and their tails were not docked. The average weaning age on F1–F4 was 27, 28, 25, and 30 days, respectively. Prior to weaning, piglets were routinely vaccinated against porcine circovirus type 2, and sows against parvovirus and erysipelas.

Pens in the growing units and finishing farms had partly slatted floors, with a space allowance according to the Finnish recommended standards: 0.4 m^2^ and 0.9 m^2^ for growing and finishing pigs, respectively (docplayer.fi/790,639-Atriasika-tuotanto-ohjeet.html). Growers on F1–F3 and all finishers were fed with a standard liquid meal, restricted in certain production phases. On F4, growers were offered a dry feed *ad libitum*. Due to a lack of space, a subset of study pigs from the growing unit of F4 was temporarily moved to an additional finishing farm owned by the same producer and located near the piglet-producing farm. This subset of pigs was transported to the final finishing farm together with the other study pigs. The piglet-producing farm F2 was a breeding unit that sold gilts as replacement stock to other farms. Therefore, the study pigs from F2 were mainly males.

### Aims, design, and setting of the study

We aimed to describe the dynamics of salivary and serum Hp, ADA, and IgG of growing pigs in four production stages (suckling, early growing, late growing and finishing), on commercial Finnish pig farms. We also investigated the association between gender and biomarker dynamics and the correlations of abovementioned biomarkers in saliva and serum at different production stages during growing. For the final analysis we included only those pigs that we could follow until the finishing age and that had not been treated due to disease symptoms during their lifetime.

The experiment took place between December 2018 and June 2019. On each piglet-producing farm, farrowing of sows in one batch were supervised for three to four days. At birth, the sex and birth weight of piglets were recorded, and all viable piglets were individually marked on their back with running numbers. Piglets weighing less than 0.9 kg at birth were excluded due to a poor prediction of survival [23] in order to retain the study population until the end of the study. On the following day, a maximum of six piglets of both sexes from each litter were ear-tagged. Out of the initially 163 ear-tagged piglets, 42 died or were medicated before the finishing stage. In addition to that, we lost track of four piglets. We selected to laboratory analyses the samples and to other analyses the data that were collected from unmedicated pigs, which we could follow until the finishing stage resulting in 117 pigs. Out of them, 48 (41.0%) were females and 69 (59.0%) males.

Individual saliva and blood samples were collected at four production stages, three times on piglet-producing farms (production stages suckling, early growing and late growing) and once in finishing farm (production stage finishing). The age of the piglets at suckling sampling was a median (min–max) of four (1–5) days, at early growing 24 (21–33) days, at late growing 66 (61–80) days and at finishing 165 (132–168) days. All male piglets were intact at the time of the first sampling. The piglets were weighed right after birth, at early growing, at late growing and at finishing.

### Sampling procedures

Whenever possible, saliva was collected prior to blood sampling. Due to the farm circumstances (e.g. if pigs were distributed between one or several rooms) and available research personnel, sampling of saliva and blood sometimes occurred in the opposite order.

### Saliva

Commercial cotton pads intended for saliva collection (Salivette®, Sarstedt, Nümbrecht, Germany) were used for saliva sampling. Individual pigs were allowed to chew the pad for a few minutes until moistened. For suckling piglets, the pad was either kept in the mouth by tying a string around the piglet´s head or held with forceps. In later samplings, the pad was always held with forceps. After sampling, the pad was placed into a sampling tube provided by the manufacturer.

### Blood

Blood was sampled by venipuncture of the jugular vein. Blood samples were taken from suckling piglets by holding them in the sampler’s arm, and those at the beginning of the growing period were taken by holding the piglet either in the sampler’s arms or on the sampler’s knees. Growers and finishers were sampled in a standing position in their pens or in the room corridor, and a snout snare was used to restrain the pigs for blood sampling.

### Processing of samples

All samples were stored at refrigerator temperature until processed within 24 h. Saliva samples were centrifuged for 10 min at 3000 x g, and blood samples for 10 min at 100 x g. The supernatant of saliva and serum samples was collected. For blood samples from suckling piglets, serum immunoglobulin ratio (immunocrit) was determined after centrifugation according to Vallet et al. [28]. All saliva samples and the rest of the serum samples were stored at -80 °C, and analyzed for Hp, ADA, and IgG.

### Laboratory analyses

Salivary and serum Hp concentrations in a 1:10 and 1:1000 dilution, respectively, were determined by using an in-house time-resolved immunofluorometric assay previously validated by Gutiérrez et al. [30]. In summary, the overall precision of the assay showed an intra-assay coefficient of variation (CV) of 1.69% and an inter-assay CV of 11.07%; the accuracy was good, with a coefficient of determination of 0.97 when linearity under dilution was analyzed, and the limit of detection was 0.52 ng/mL. A previously optimized adaptation of a commercial automatized assay (BioSystems S.A.) [28] was used to determine salivary and serum ADA activity levels (ΔOD/min x 3333 = U/L) according to the manufacturer’s instructions. For the ADA quantification, saliva was diluted 1:16 or if extremely high ADA values were obtained, 1:32. Serum was analyzed undiluted for ADA determination. The overall intra-assay CV was 4.71% and the coefficient of determination of 0.98, indicating good accuracy. The limit of detection was 9.33 U/L ( [28]. The inter-assay precision for salivary ADA determinations was calculated internally and showed an overall CV of 8.01 (data not published). A commercial ELISA kit (Pig IgG ELISA kit, Bethyl Laboratories Inc., Montgomery, USA) was used according to the manufacturer’s instructions to determine the IgG concentration in saliva samples and the serum IgG concentration, except for suckling piglets. Validation of the salivary IgG assay has been reported by Escribano et al. [27]. In brief, the analytical validation of the assay yielded overall intra-assay and inter-assay CVs of 3.66% and 7.81%, respectively, indicating good accuracy with a coefficient of determination of 0.99 and a limit of detection of 7.74 ng/mL. The validation of the kit for the serum IgG measurement was performed internally (data not published) and demonstrated an overall intra-assay precision of 9.87% and an inter-assay precision of 13.97%. The median coefficient of determination after the study of the linearity under dilution in two serum samples was 0.98 and the limit of detection was 7.81 ng/mL. Saliva was diluted 1:500 prior to the IgG analysis and in the case of extremely high IgG values, a 1:700 dilution was used. Serum was analyzed at a dilution of 1:500 000 for IgG determination.

To determine the serum immunoglobulin ratio, 50 µL of serum was mixed with 50 µL of a 40% solution of ammonium sulfate. The mixture was absorbed into a hematocrit capillary that was sealed at its other end with a hematocrit sealer. The capillaries were loaded into a hematocrit centrifuge and centrifuged for 5 min at 12 700 x g. The precipitate length and the length of a precipitate plus liquid layer were measured with a ruler, and their ratio calculated according to the following formula:

$$ \begin{aligned} & piglet\;serum\;immunoglobulin\;ratio \\ & \quad = \frac{precipitate\;length\;\left(mm\right)}{precipitate\;length + length\;of\;the\;liquid\;layer\;\left(mm\right)} \end{aligned}$$

The serum immunoglobulin ratio of 0.1 corresponds to an IgG concentration of 34 mg/mL. Thus, being an estimate, the conversation of the serum immunoglobulin ratio to IgG concentration was only used in the descriptive part of the results to better reflect the differences between the different stages of production over the whole study period.

### Statistical analyses

SPSS (IBM SPSS Statistics for Windows, Version 25.0, Armonk, NY; IBM Corp.) was used for statistical analyses. The normality of all continuous variables was checked visually and with the Shapiro-Wilk test. As both saliva and serum HP, as well as serum IgG were non-normally distributed log10-transformations were used to achieve normality. Descriptive statistics are presented based on raw data due to the transformation of some of the variables. Pig was the experimental unit in all statistical analyses.

Independent t-tests in each age group were used to assess whether weight of the study pigs differs between genders.

To investigate the dynamics of the selected biomarkers across four stages of production, repeated-measures linear mixed models (LMM) were constructed. Each biomarker, both in saliva and in serum, was set separately as dependent variable, resulting in six models altogether. All the models included pig as the subject and production stage as repeated variable. For the model for serum IgG, measures were available from early growing stage, whereas all the other models included measurements from all stages of production. The initial models contained gender, production phase, and their interaction as fixed variables, pig weight at the time of sampling as a covariate, and sow nested within farm as a random variable. We then used a backward elimination procedure, removing non-significant variables from the models step by step, using the Akaike information criterion (AIC) to decide on the best model. Production stage and gender were, however always kept in the models. Pairwise comparisons between production phases were run with a Bonferroni correction. Models were assessed based on the normality of their residuals.

Non-parametric Spearman correlations were computed using all pigs between each biomarker and in both body fluids, and separately in all four stages of production. The Spearman correlation coefficients (ρ) were interpreted as strong (> 0.6), moderate (0.4–0.59), weak (0.2–0.39), or very weak (< 0.2) according to [31].

Sample size needed was calculated by using Epitools two means with unequal sample size and unequal variances calculation to achieve a power of 0.8 and a confidence level of 0.05. Sample size calculations resulted that 47–71 pigs were needed for the study to reveal statistical differences between biomarker concentrations at different times, depending on the biomarker. Assumptions were given in the calculations as mean (sd) for different biomarkers: ADA 500 (150) U/L vs. 600 (250) U/L, Hp 2.0 (1.5) mg/mL vs. 1.4 (0.9) mg/mL and IgG 8.0 (3.0) mg/mL vs. 10.0 (4.0) mg/mL.

### Electronic supplementary material

Below is the link to the electronic supplementary material.

Supplementary Material 1

## Data Availability

The datasets used and/or analyzed during the current study are available from the corresponding author on reasonable request.
