# Supplementary material for: Salivary and serum haptoglobin, adenosine deaminase, and immunoglobulin G in growing pigs
Source: Porcine Health Manag. 2024 May 21;10:21. doi: 10.1186/s40813-024-00368-8 (PMC11110307; doi:10.1186/s40813-024-00368-8)
Supplement: Supplementary file 1 — Supplementary Material 1 [file 40813_2024_368_MOESM1_ESM.docx]

Supplementary Tables

Table 1a Final output of the linear mixed model with repeated measures for porcine salivary haptoglobin.

.

|  | df | F | p-value |
| --- | --- | --- | --- |
| Intercept | 1.36 | 41.6 | < 0.001 |
| Production Stage^1)^ | 3.133 | 250.0 | < 0.001 |
| Gender | 1.179 | 2.3 | 0.13 |
| Production Stage*Gender ^2)^ | ^3)^ |  | ^3)^ |
| Weight | ^3)^ |  | ^3)^ |

The dependent variable was Log10 –transformed for the analysis and number of subjects (pigs) in the model was 91. ^1)^ Saliva was sampled at suckling, early growing, late growing and finishing stages. ^2)^ Interaction. ^3)^ not included in final model for salivary haptoglobin.

Table 1b Final output of the linear mixed model with repeated measures for porcine salivary adenosine deaminase.

|  | df | F | p-value |
| --- | --- | --- | --- |
| Intercept | 1.87 | 186.8 | < 0.001 |
| Production Stage^1)^ | 3.123 | 50.5 | < 0.001 |
| Gender | 1.254 | 5.6 | 0.018 |
| Production Stage*Gender^2)^ | ^3)^ | ^3)^ | ^3)^ |
| Weight | 1.86 | 5.0 | 0.028 |

Number of subjects (pigs) in the model was 90. ^1)^ Saliva was sampled at suckling, early growing, late growing and finishing stages. ^2)^ Interaction. ^3)^ not included in final model for salivary adenosine deaminase.

Table 1c Final output of the linear mixed model with repeated measures for porcine salivary immunoglobulin G.

|  | df | F | p-value |
| --- | --- | --- | --- |
| Intercept | 1,91 | 439.4 | < 0.001 |
| Production Stage^1)^ | 3.05 | 78.7 | < 0.001 |
| Gender | 1.186 | 1.1 | 0.28 |
| Production Stage*Gender ^2)^ | ^3)^ | ^3)^ | ^3)^ |
| Weight | 1,80 | 8.6 | 0.004 |

The dependent variable was Log10 –transformed for the analysis and number of subjects (pigs) in the model was 90. ^1)^ Saliva was sampled at suckling, early growing, late growing and finishing stages. ^2)^ Interaction. ^3)^ not included in final model for salivary immunoglobulin G.

Table 1d Final output of the linear mixed model with repeated measures for porcine serum haptoglobin.

|  | df | F | p-value |
| --- | --- | --- | --- |
| Intercept | 1,42 | 72.7 | < 0.001 |
| Production Stage^1)^ | 3,183 | 7.2 | < 0.001 |
| Gender | 1,376 | 0.07 | 0.80 |
| Production Stage*Gender ^2)^ | ^3)^ | ^3)^ | ^3)^ |
| Weight | ^3)^ | ^3)^ | ^3)^ |

The dependent variable was Log10 –transformed for the analysis and number of subjects (pigs) in the model was 117. ^1)^ Serum was sampled at suckling, early growing, late growing and finishing stages. ^2)^ Interaction. ^3)^ not included in final model for serum haptoglobin.

Table 1e Final output of the linear mixed model with repeated measures for porcine serum adenosine deaminase.

|  | df | F | p-value |
| --- | --- | --- | --- |
| Intercept | 1.375 | 4968.2 | < 0.001 |
| Production Stage^1)^ | 3.214 | 9.5 | < 0.001 |
| Gender | 1.375 | 1.7 | 0.20 |
| Production Stage*Gender ^2)^ | 3.215 | 2.2 | 0.090 |
| Weight | ^3)^ | ^3)^ | ^3)^ |

Number of subjects (pigs) in the model was 117. ^1)^ Serum was sampled at suckling, early growing, late growing and finishing stages. ^2)^ Interaction. ^3)^ not included in final model for serum adenosine deaminase.

Table 1f Final output of the linear mixed model with repeated measures for porcine serum immunoglobulin G.

|  | df | F | p-value |
| --- | --- | --- | --- |
| Intercept | 1.40 | 708.6 | < 0.001 |
| Production Stage^1)^ | 2.195 | 246.5 | < 0.001 |
| Gender | 1.295 | 3.6 | 0.060 |
| Production Stage*Gender ^2)^ | 2.195 | 5.0 | 0.008 |
| Weight | ^3)^ | ^3)^ | ^3)^ |

Number of subjects (pigs) in the model was 117. ^1)^ Serum was sampled at early growing, late growing and finishing stages. ^2)^ Interaction. ^3)^ not included in final model for serum adenosine deaminase.

Table 2a Descriptive statistics of three porcine salivary biomarkers by gender and four production stages.

|  | | **Female**  **Median** | **IQ** | **n** | **Male**  **Median** | **IQ** | **n** |
| --- | --- | --- | --- | --- | --- | --- | --- |
| **Suckling,**  **4 (1-5) days** |  | |  |  |  |  |  |
| Hp (μg/mL) | 8.58 | | 4.01 | 27 | **7.49** | 4.93 | 29 |
| ADA (U/L) | 878.25 | | 421.96 | 38 | **928.57** | 352.63 | 37 |
| IgG (μg/mL) | **627.33** | | 990.81 | 21 | 315.61 | 282.53 | 17 |
| **Early growing, 24 (21-33) days** |  | |  |  |  |  |  |
| Hp (μg/mL) | 1.69 | | 1.83 | 37 | **2.42** | 1.76 | 41 |
| ADA (U/L) | 603.27 | | 367.30 | 40 | **621.27** | 427.96 | 45 |
| IgG (μg/mL) | 36.84 | | 110.78 | 39 | **40.04** | 49.21 | 44 |
| **Late growing, 66 (61-80) days** |  | |  |  |  |  |  |
| Hp (μg/mL) | 0.87 | | 2.27 | 35 | **1.11** | 2.04 | 33 |
| ADA (U/L) | 308.97 | | 236.48 | 36 | **417.96** | 407.96 | 37 |
| IgG (μg/mL) | 11.05 | | 22.38 | 35 | **16.23** | 26.35 | 37 |
| **Finishing, 165 (132-168) days** |  | |  |  |  |  |  |
| Hp (**μg/mL)** | 0.31 | | 0.32 | 38 | **0.44** | 0.52 | 40 |
| ADA (U/L) | 502.62 | | 283.64 | 38 | **587.94** | 230.31 | 39 |
| IgG (**μg/mL)** | 13.74 | | 6.99 | 38 | **15.62** | 14.34 | 40 |

Hp = haptoglobin, ADA = adenosine deaminase, in 1:16 dilution, IgG = immunoglobulin G. **Age is presented as median (minimum-maximum).** Median values in bold indicate which sex measured the higher concentration at that production stage. IQ = interquartile range. n = number samples, differences in numbers are due to differences in the available analysis results of different biomarkers at each stage of production.

Table 2b Descriptive statistics of three porcine serum biomarkers by gender and through four production stages.

|  | **Female**  **Median** | **IQ** | **n** | **Male**  **Median** | **IQ** | **n** |
| --- | --- | --- | --- | --- | --- | --- |
| **Suckling,**  **4 (1-5) days** |  |  |  |  |  |  |
| Hp (mg/mL) | 0.35 | 0.91 | 40 | **0.37** | 1.09 | 53 |
| ADA (U/L) | 493.28 | 138.49 | 40 | **519.28** | 180.65 | 55 |
| IgG (mg/mL)**^1)^** | 42.69 | 11.39 | 42 | **47.72** | 14.80 | 61 |
| **Early growing, 24 (21-33) days** |  |  |  |  |  |  |
| Hp (mg/mL) | **0.17** | 0.75 | 48 | 0.10 | 0.72 | 69 |
| ADA (U/L) | 571.61 | 229.98 | 48 | **599.27** | 176.65 | 69 |
| IgG (mg/mL) | **5.12** | 2.05 | 47 | 5.08 | 2.69 | 68 |
| **Late growing, 66 (61-80) days** |  |  |  |  |  |  |
| Hp (mg/mL) | **1.08** | 1.24 | 48 | 0.76 | 1.09 | 66 |
| ADA (U/L) | 446.62 | 157.98 | 48 | **523.28** | 166.98 | 67 |
| IgG (mg/mL) | **6.43** | 2.86 | 48 | 5.49 | 2.44 | 67 |
| **Finishing, 165 (132-168) days** |  |  |  |  |  |  |
| **Hp (mg/mL)** | **0.39** | 0.53 | 47 | 0.37 | 0.53 | 66 |
| ADA (U/L) | **491.28** | 196.65 | 47 | 441.62 | 122.49 | 66 |
| IgG (mg/ml) | **10.90** | 2.78 | 47 | 9.03 | 3.26 | 67 |

Hp = haptoglobin, ADA = adenosine deaminase, IgG = immunoglobulin G. Age is presented as median (minimum-maximum). **^1)^** Estimation, which corresponds to serum immunoglobulin ratio of 0.125 (0.039) in females and 0.137 (0.044) in males. Median values in bold indicate which sex measured the higher concentration at that production stage. IQ = interquartile range. n = number samples, differences in numbers are due to difference in the available analysis results of different biomarkers at each stage of production.
